# High Hydrostatic Pressure to Increase the Biosynthesis and Extraction of Phenolic Compounds in Food: A Review

**DOI:** 10.3390/molecules27051502

**Published:** 2022-02-23

**Authors:** Jorge E. Navarro-Baez, Luz María Martínez, Jorge Welti-Chanes, Génesis V. Buitimea-Cantúa, Zamantha Escobedo-Avellaneda

**Affiliations:** Escuela de Ingeniería y Ciencias, Tecnologico de Monterrey, Eugenio Garza Sada 2501, Monterrey 64700, Mexico; a00820540@itesm.mx (J.E.N.-B.); luzvidea@tec.mx (L.M.M.); jwelti@tec.mx (J.W.-C.); genesis.vidal@tec.mx (G.V.B.-C.)

**Keywords:** phenolic compounds, high hydrostatic pressure, reactive oxygen species, biosynthesis, extraction, stress response, cell wall modification

## Abstract

Phenolic compounds from fruits and vegetables have shown antioxidant, anticancer, anti-inflammatory, among other beneficial properties for human health. All these benefits have motivated multiple studies about preserving, extracting, and even increasing the concentration of these compounds in foods. A diverse group of vegetable products treated with High Hydrostatic Pressure (HHP) at different pressure and time have shown higher phenolic content than their untreated counterparts. The increments have been associated with an improvement in their extraction from cellular tissues and even with the activation of the biosynthetic pathway for their production. The application of HHP from 500 to 600 MPa, has been shown to cause cell wall disruption facilitating the release of phenolic compounds from cell compartments. HPP treatments ranging from 15 to 100 MPa during 10–20 min at room temperature have produced changes in phenolic biosynthesis with increments up to 155%. This review analyzes the use of HHP as a method to increase the phenolic content in vegetable systems. Phenolic content changes are associated with either an immediate stress response, with a consequent improvement in their extraction from cellular tissues, or a late stress response that activates the biosynthetic pathways of phenolics in plants.

## 1. Introduction

Phenolics are a group of specialized metabolites with antioxidant, antimicrobial, anticancer, anti-inflammatory activity, among other biological properties for human health [1,2]. Fruits and vegetables are rich sources of these compounds. Due to their association with treating and preventing some chronic diseases, a diet rich in vegetables and fruits is highly recommended [3,4].

Thermal treatments are frequently used during fruit and vegetable processing to inactivate pathogenic and spoilage microorganisms and enzymes to ensure food safety and quality [5]. However, these treatments can decrease the nutritional value by reducing thermosensitive bioactive compounds, along with modifying texture, taste, and flavor [5,6]. To overcome these adverse effects, other alternatives such as high hydrostatic pressure (HHP) have been used to preserve food. HHP consists of applying pressures normally up to 600 MPa into a chamber. The pressure is generally transmitted by water at room or moderate temperature. This treatment can inactivate microorganisms and enzymes while minimizing quality losses in the pressurized products [7]. This technology is considered an innovative technique for the nonthermal treatment of food [8].

Pressure is a physical parameter that affects the state of physical, chemical, and biological systems. Pressure can modify the chemical configuration of some food molecules changing the rate of chemical and enzymatic reactions [9]. HHP is governed by the Le Chatelier and Pascal principles [10]. The former states that a system under pressure will adopt molecular configurations and will adjust the rate of chemical reactions to achieve the lowest volume [11]. In other words, pressure favors phenomena and reactions that are accompanied by negative volume changes [12]. While the Pascal or isostatic principle states that the pressure applied is instantly and uniformly transmitted in all directions of the fluid and its surroundings [13].

HHP to inactivate microorganisms was first proposed in the late 1980s. Since then, the use of this technology as a preservation method has increased in the food industry. The ability of HHP to inactivate microorganisms and enzymes related to food oxidation are some examples of the benefits involved [7]. HHP-treated products present better nutrient retention, flavor, and color. Moreover, HHP reduces or eliminates the use of additives and does not produce residues during processing because only water is involved in the entire process [14,15,16]. Nevertheless, some enzymes and bacterial spores are highly resistant to pressure requiring the combination of pressure and temperature for their inactivation. In addition, some residual enzyme activity and dissolved oxygen could cause enzymatic and oxidative degradation of specific food components, and most HHP-treated products need to be stored at low temperature [17]

In addition to food preservation, HHP has been proposed as a method to enhance bioactive compound content in foods and to improve their extraction. Some pressurized foods have shown higher contents of functional compounds such as phenolics compared with untreated products. The increments have been associated with the release of these compounds from cellular compartments, resulting in increased extractability due to the mechanical stress occurring during the pressurization, which compacts the cellular morphology, cell wall, and organelles [18]. Another mechanism for the increment is related to the immediate response of plants to generate signaling molecules that activate pathways that regulate gene expressions or with the late response associated with higher enzyme activity produced by the signaling molecules generated in the immediate response, which activate the biosynthesis of specialized metabolites [19].

This review analyzes the mechanisms for phenolic increment in vegetables and fruits immediately after HHP treatment and during storage, differentiating between increment due to cellular disruption (improvement of extraction yield) or activation of the metabolic pathways for compound biosynthesis.

## 2. Biosynthesis of Phenolics in Plants

**Figure 1:**
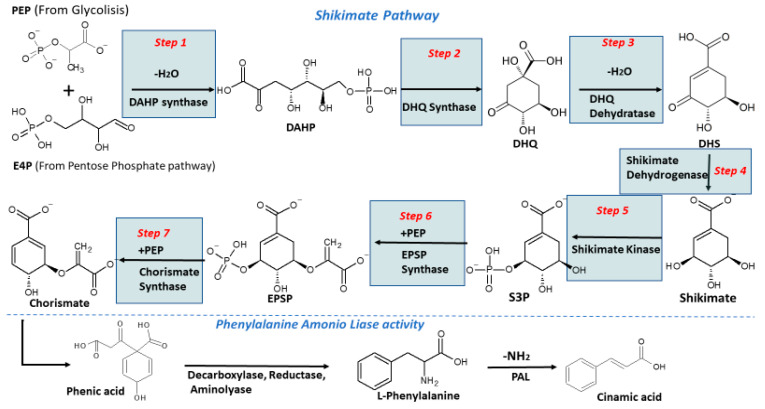
Shikimate pathway for the production of phenolics in plants. PEP: phosphoenolpyruvate, E4P: erythrose-4-phosphate, DAPH: 3-deoxy-D-arabinoheptulosonate 7-phosphate, DHQ: 3-dehydroquinate, S3P: shikimate 3-phosphate, EPSP: 5-enolpyruvylshikimate 3-phosphate, PAL: Phenylalanine Ammonium Lyase. Modified from [22,31].

The biosynthesis of phenolic in plants is achieved by the malonate and the shikimate pathways [20]. The malonate pathway is mainly focused on the synthesis of secondary fatty acids, and some phenolic compounds such as aromatic polyketides, where flavonoids are included. The precursors of this pathway are acetyl-CoA and malonyl-CoA [21]. The shikimate pathway (Figure 1) is responsible for the biosynthesis of most phenolics in plants [22]. This consists of seven steps carried out by different enzymes, and starts with two molecules, erythrose-4-phosphate (E4P) and phosphoenolpyruvate (PEP) derived from glycolysis and pentose phosphate pathways, respectively [23]. The first step consists of the condensation of erythrose-4-phosphate (E4P) and phosphoenolpyruvate (PEP) into 3-deoxy-D-arabinoheptulosonate 7-phosphate (DAHP) by the DAHP synthase [24]. In the second step, the DAHP is cyclized, forming 3-dehydroquinate (DHQ) due to the action of DHQ synthase [25]. In the third and fourth steps, DHQ is dehydrated to 3-dehydroshikimate (DHS) and then is reduced to shikimate by the bifunctional enzyme 3-dehydroquinate dehydratase/shikimate dehydrogenase [26,27]. In the fifth step, the shikimate is converted to shikimate 3-phosphate (S3P) by the shikimate kinase [28]. In the sixth step, the shikimate 3-phosphate is condensate with a second molecule of PEP into 5-enolpyruvylshikimate 3-phosphate (EPSP) [29]. Finally, the EPSP is dephosphorylated by the chorismate synthase to produce chorismite [30].

After the production of chorismate, the phenic acid is formed by the enol-pyruvate transferase action, the phenic acid goes under a decarboxylation, and the substitution of the oxygen for an amino group results in the formation of L-phenylalanine [31]. The most important step occurs by the phenylalanine ammonium lyase (PAL). This enzyme deaminates the L-phenylalanine into cinnamic acid. L-phenylalanine is a block for the formation of secondary metabolites, so its relationship with the biosynthesis of phenolic compounds is important [31,32,33] (Figure 1). The production of phenolic compounds in plants is strictly related to the activity of the enzyme PAL. A study in strawberries showed that at the peak of maximum PAL activity, there was a higher anthocyanin content [34]. The formation of metabolites such as phenolic compounds is also related to the defense mechanism against biotic or abiotic stress [32].

### Biotic and Abiotic Factors Influencing Biosynthesis of Phenolics

The production of specialized metabolites in plants is affected by biotic and abiotic factors, such as environmental conditions, microorganisms, insect attacks, among others [35]. Biotic stress is caused by the action of bacteria, fungi, viruses, and nematodes that attack the plants by secreting enzymes to break down tissues. Insects and vertebrates are also biotic stressors that use plants as a food source [36]. Abiotic stress is caused by external factors such as drought, soil salinization, extreme temperatures, strong winds, climate, and change of season of the year [36]. Even air pollution and the use of pesticides can act as abiotic factors [35].

Phenolic compounds are implicated in the biotic and abiotic stresses by reinforcing cell walls and scavenging of ROS (Reactive Oxygen Species) [37]. Biotic and abiotic stressors promote the production of free radicals and oxygen species in plants, inducing the synthesis of secondary products like phenolics as a mechanism to protect plants [38]. Some researchers have taken advantage of this to seek new ways to deliberately increase bioactive compounds in plants. Some techniques to increase phenolics in plants are based on controlled elicitation. One example is the use of nanoparticles (NPs) such as Cu, CdO, CeO2, CuO, Ag, and ZnO as abiotic elicitors for induction of phenolic and other bioactive compounds in plant cells [39]. NPs induce the production of ROS, leading to the transcription of secondary metabolites [40]. The effect on NPs as elicitors vary, but generally shows an increment of secondary metabolites such as phenols and flavonoids [41]. However, an important drawback of the use of NPs is their toxicity [42]. It has been suggested that the elevated production of ROS by NPs results in lipid peroxidation, which damages cell membrane, proteins, and DNA resulting in cell death [43].

Mechanical force is another important abiotic stress factor that has shown a positive effect on the production of phenolics in plants. HHP at pressure levels below 100 MPa acts as a mechanical stressor resulting in an increment of phenolic levels [44,45]. Pressure, temperature, and time of exposure are variables that have been studied for the biosynthesis of metabolites in plants under HHP [9]. A very useful advantage of this technology is the retention of cell viability at certain treatment conditions, which allows the cells to keep enzymatic activities to induce significant production of phenolics [44]. The HHP potential for the biosynthesis of phenolic compounds is presented in the following sections, differentiating it from the increment due to improvement in extraction yields.

## 3. HHP as a Stress Factor for the Biosynthesis of Phenolics and to Increase Their Extraction Yield

### 3.1. Effect of HHP on Phenolics Biosynthesis

Although the effects of HHP on the biosynthesis of phenolics have been evaluated in fruits and vegetables such as mangoes, carrots, strawberries, and suspension cultures of grapes and potato, there is not yet enough research on the mechanism(s) implied in the increment for the biosynthesis of phenolics by HHP. Several studies using pressures from 10 to 100 MPa at treatment times from 10 to 20 min at room temperature have been tested immediately after processing or during the storage at different temperatures and relative humidities; and the results have revealed a change in phenolics biosynthesis with increments up to 155%. Some studies conclude biosynthesis of phenolics due to the low-pressure levels used and the increment in their content, nevertheless, some of them do not show studies related to increment in PAL activity, ROS production, or gene expression, which are important to conclude this (Table 1).

**Figure 2:**
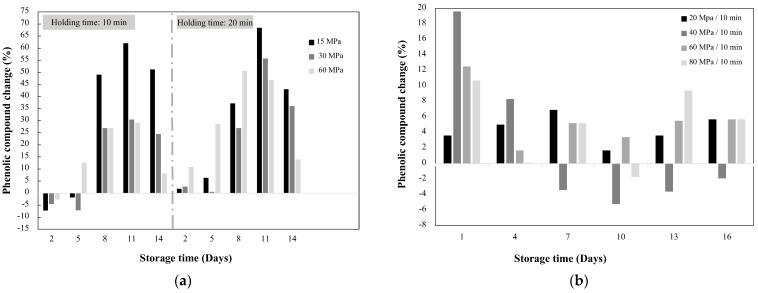
Effect of high hydrostatic pressure in the increment of phenolic compounds of mango *(Mangifera indica)* stored at two conditions. (**a**) Fruits stored at 25 ± 1 °C with 85–90% relative humidity [46] and (**b**) fruits stored at 13 °C with ~85% humidity [46].

Phenolic biosynthesis due to HHP does not seem to increase proportionally with the pressure. The increments primarily depend on the type of fruit treated and the ripening stage, as well as, the storage conditions such as temperature, relative humidity, and storage time [46,47]. In addition, it has been observed that the increment does not occur immediately after processing, but during storage producing a late stress response. For example, mango treated at 60 MPa/20 min showed an increment in phenolics of about 11, 29, and 47% after two, five, and eight days of storage, respectively, compared to nontreated samples (Figure 2a) [46]. After day eight, the concentration of phenolics starts to decay [46,48]. This decrement could be associated with the activity of oxidative enzymes, which are normally inactivated at higher pressure levels (>200 MPa) than the ones used to induce stress in the vegetable systems. According to Ortega et al. [48], the initial improvement in phenolic content could be attributed to an increment in their biosynthesis due to immediate oxidative stress, while reductions at longer times could be related to the damage in cellular structures and the ripening process [46]. In another study with mango, Hu et al. [47] showed 19.6% increment in phenolics after one day of storage (Figure 2b) and 69.7% increment in flavonoids after four days. Overall, Ortega et al. [46] found greater increments in phenolics compared to Hu et al. [47], which could be attributed to differences in treatment conditions, ripening stage of the fruit, and the storage conditions. It has been proved that 25 °C is the best temperature for mango ripening, which may result in improved phenolic compounds and other metabolites such as organic acids and sugars [51]. Both studies agreed that pressures around 60 and 80 MPa have an initial increase in phenolics, but the concentration gradually decreases with storage time. Although both studies suggest biosynthesis of phenolic compounds due to HHP; the authors did not show any test to probe the biosynthesis, for example, the increment in PAL activity. Further studies need to be performed to prove the biosynthesis of phenolic compounds [32,50].

**Figure 3:**
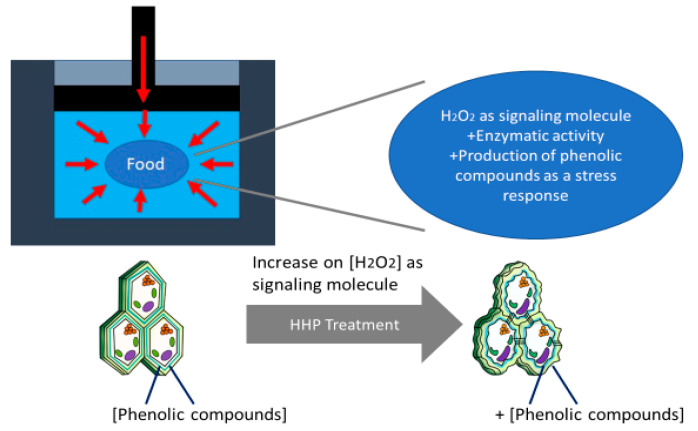
HHP effect in cell wall deformation from food. Modified from Gómez-Maqueo, et al. [18].

Ortega et al. [46] suggested that pressure as an abiotic stressor can lead to cell wall fracture or deformation causing cell wall loosening by crosslinking or depolymerizing its components [52]. Plant cells can sense the mechanical perturbation at their cell surfaces and they respond [53]. This promotes the production of ROS, like H_2_O_2_, which later acts, controls, and initiates enzymatic responses to repair the damaged cell wall via stress-responsive gene, oxidative burst linked with cell wall reinforcement, biosynthesis of phenolics, among others [9,48,54]. Injuries caused by mechanical stress, such as pressure, modify how plants synthesize secondary metabolites, as can be represented in Figure 3 [35]. The release of H_2_O_2_ is carried out in minutes, acting as the elicitor in the biosynthesis. The production of H_2_O_2_ at the cellular level acts as Ca+2 signaling, activates kinases, hormonal signaling, and regulates gene expression [54,55]. H_2_O_2_ as a signaling molecule activating metabolic pathways [56], leads to increased PAL activity, which as previously mentioned, synthesizes simple phenols derived from the cinnamic acid [19,31,32].

**Figure 4:**
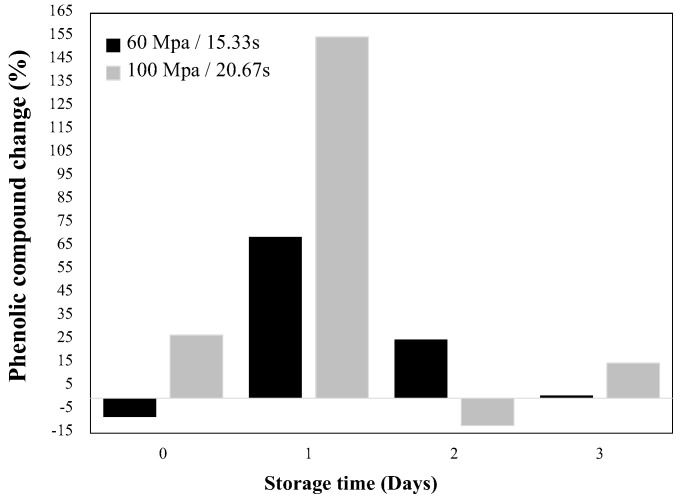
Effect of HHP in the biosynthesis of phenolic in carrots (*Daucus carota)*. Adapted from Viacava et al. [49].

**Figure 5:**
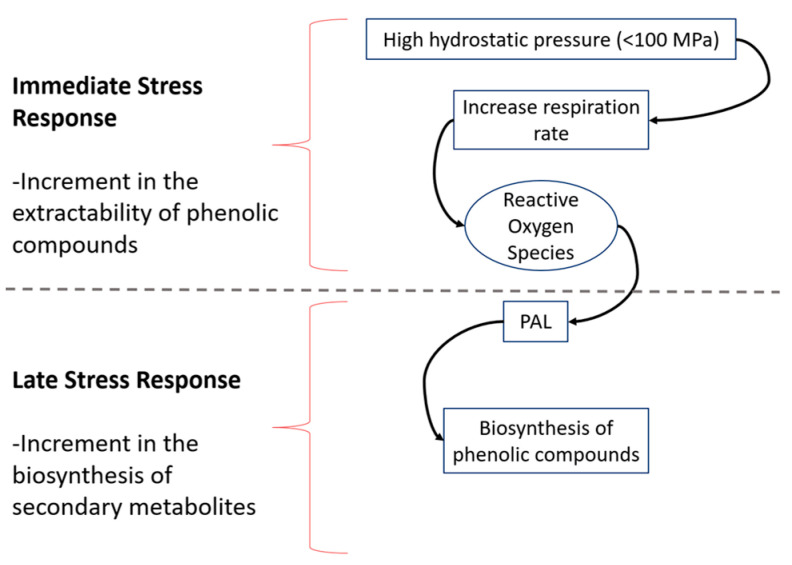
Immediate and late physiological response to stress produced by HHP technology to enhance extractability and biosynthesis in phenolic compounds in plants. Modified from [49].

Similar to mango, for HHP-treated carrots after one day of storage, the total phenolic content increased 69.1% at 60 MPa and 154.9% at 100 MPa [49]. After day one, phenolic concentration starts to decrease showing similar values to the control (Figure 4). Viacava et al. [49], related the change in phenolic concentration with PAL activity observing an immediate effect in PAL. On day 0, PAL activity was reduced by 61.4% (100 MPa), while no significant increment was presented at day 1 at any pressure level evaluated; however, it increased at day 2 by 380.2 and 139.7% at 60 and 100 MPa, respectively. The authors attributed the low activity of PAL at day 1 and the higher concentrations of phenolic content on the same day, to the higher availability of precursors during the HHP treatment and the activation of enzymes not quantified in the study. In general, results showed that the PAL activity was higher at 60 than at 100 MPa, having greater metabolic activity because the samples were under higher oxidative stress-producing higher ROS, which resulted in higher PAL activity. It has shown that pressure has different effects on enzyme activities depending on factors such as type of product, type of enzyme, and treatment conditions. It has been stated that pressure could have favorable effects on the release of membrane-bound enzymes, or in the activation of proenzymes that require a biochemical change or a change on their configuration to expose the active site and to become active, and a direct relationship is not always observed between the increment in enzyme activity and pressure level [57]. Figure 5 shows the hypothetical model from Viacava et al. [49] explaining the immediate and late physiological response of carrots to HHP application, this hypothetical model could be applied to other vegetables and fruits. The immediate response involves cell wall deformation (mass exchange), increment in respiration rate, and production of signaling molecules such as H_2_O_2_, while the late response involves the biosynthesis of secondary metabolites during storage [18,19,47].

The synthesis of phenolics in cell cultures has also been evaluated. Cai et al. [48] studied the synthesis of anthocyanins during seven days of storage of a cell suspension of Vitis vinifera treated at 40 MPa for 10 min, observing the greatest increment at day 6 (53.3%). For potato (*Solanum tuberosum*) suspension culture treated at 100–200 MPa for 10 min, phenolic content increased immediately after processing by 54, 81, 267, 456, and 453% at pressures of 100, 125, 150, 175, and 200 MPa, respectively; nevertheless, these increments appear not to be associated with biosynthesis, but rather with the loss of compartmentation and subsequent release of the content of the vacuoles into the cytoplasm [44].

Bioactive compounds, such as phenolics, are contained in specific organelles in the cell, which can vary depending on each type of product and variety [18]. The increment in phenolic compounds observed after HHP is not always attributed to biosynthesis. The release of phenolics by extraction from specific organelles could be responsible for the increments. The better extractability of phenolic at pressure levels above 100 MPa has been related to cell membrane disruption and release of bound phenolics, resulting in higher extractability and an improvement in bioaccessibility [58]. Making it different from biosynthesis, which is suggested to be a dual stress-response mechanism related to ATP, ROS, and the activation-deactivation of enzymes [18,19,47,50]. In the following section, some studies showing an increment of phenolics due to improvement of extraction are discussed.

### 3.2. Effect of HHP on Phenolics Extraction Yield

Several methods have been applied for phenolic extraction, such as conventional solvent extraction (CSE), and novel technologies like ultrasonic-assisted extraction (UAE), microwave-assisted extraction (MAE), and supercritical fluid extraction (SFE-CO_2_) [59,60,61]. The application of novel technologies has resulted in favorable results on extraction yields compared with CSE [60,61]. 

HHP can also favor the release of bioactive compounds from cellular compartments, enhancing their extractability [18]. When fruits and vegetables are subjected to pressure levels normally higher than 100 MPa, the mechanism for phenolic biosynthesis is not promoted because the cell is inactivated before reacting to the stress caused by pressure [18]. In this case, the increment observed in phenolic can be attributed to improvement in extraction rather than to biosynthesis. HHP increases mass transfer in an immediate response due to the damage caused to the cell membrane, which increases permeability and facilitates secondary metabolites diffusion via solvent extraction [59,62]. In addition, disruption of weak interaction between phenolics and cell wall favors their release. In plants, phenolic compounds exist in both free and bound forms [60]. In dry fruits, bound phenolic content (mgGAE/100 g) ranged from 96 to 408; and free phenolic from 46 to 345. While in fresh fruits, the bound phenolic content ranged from 29 to 306; and free phenolics from 120 to 316 [63]. The main difference between free and bound phenolics is that free is solvent extractable, while the bound phenolic cannot be extracted into water or aqueous/organic solvents mixtures [60]. Based on this information, it is suggested that during the application of HHP treatments, the increment in free phenolic content would be attributed to the cell decompartmentalization, which produces phenolic release from plant tissue improving yield extraction, while the increment of bound phenolic compounds in addition to decompartmentalization is probably due to the increment of the enzyme’s activity involved in the hydrolysis of proanthocyanidins, phenolic acids, and hydrolyzable tannins, which are esterified-bound and glycosylated-bound [61].

**Figure 6:**
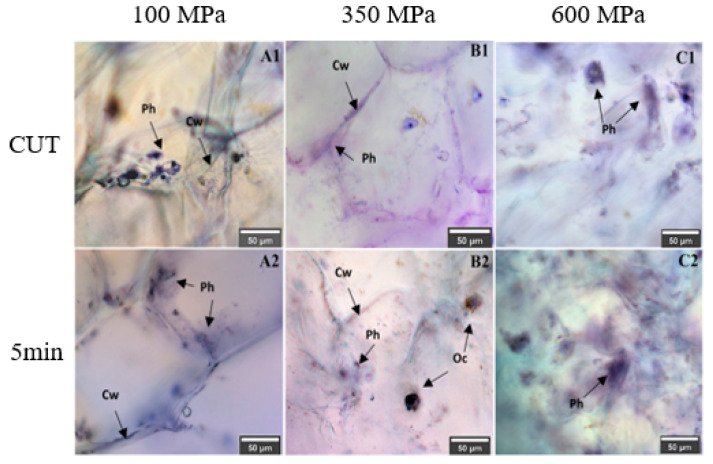
Optical microscopy images showing the effects of HHP in phenolics extractability at 100 (**A**), 350 (**B**), and 600 (**C**) MPa during the come-up time (CUT) and 5 min. Cw: cell wall, Ph: phenolic compound, Oc: calcium oxalate crystal. Modified from [18].

The effects on plant tissues, organelles, cell walls, and membranes, depend on the pressure level [62]. For example, in a study with prickly pears [18], the application of HHP at 100 MPa helped to release phenolics attached to cell walls by cell wall modifications (Figure 6). At this pressure level, the cell was still viable and capable to synthesize metabolites in response to the abiotic stress, but at higher pressure levels (350–600 MPa), the cell wall collapsed, enhancing the extractability of phenolic at pressures higher than 100 MPa. Higher pressure levels, and times, favored the extraction of phenolics due to the higher loss of cell wall integrity [18]. Table 2 shows studies for a variety of foods (vegetables, fruits, by products of plants, etc.) in which the authors have observed increment in phenolics (anthocyanins, flavonoids, polyphenols, and individual phenolics), suggesting improvement in their extractability at treatment conditions mainly from 300–600 MPa for 5–20 min at temperatures generally around 20–40 °C.

**Figure 7:**
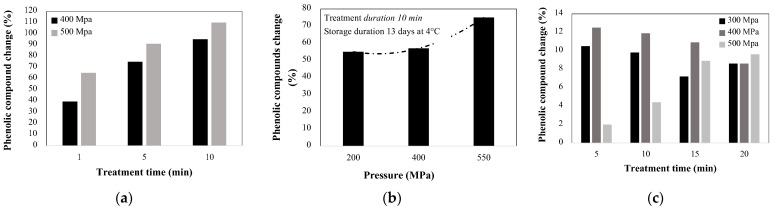
HHP effect in the extraction of phenolic compounds from (**a**) Sour cherry pomace *(Prunus cerasus* L.) [65], (**b**) Grape (*Vitis vinifera*) [69], and (**c**) apricot nectar (*Prunus armeniaca* L.) [64].

In most cases presented in Table 2, HHP showed a positive effect on the extraction of phenolics. Results indicate that the higher the pressure and treatment time, the higher the extractability of phenolics. Some remarkable results are from Okur et al. [65] for sour cherry (Figure 7a), in which the increment in time of treatment from 1 to 10 min enhances the extraction yield from 39.5 up to 95% at 400 MPa and from 65 up to 109.9% at 500 MPa, respectively. Also, the increment in pressure from 400 to 500 MPa resulted in improvement of the extracted phenolics from 39.5 up to 61% [65]. The same tendency was shown for grape (Figure 7b), where the extraction of phenolic increased from 55 up to 75% by increasing the pressure treatment from 200 to 550 MPa [69]; gooseberry pulp treated at 400 and 500 MPa (during 10 min) showed an increment in the extracted phenolics of 8.3 and 22.9%, respectively [68]. In other studies, the treatment at 500 MPa of apricot nectar, showed an increment in total phenolics from 2 up to 9.6% when the holding time increases from 5 to 20 min (Figure 7c) [64]. Liu et al. [76] in wild berry demonstrated that at 200 MPa, the anthocyanin content increased from 6.3 to 8% by increasing the holding time from 5 to 10 min. 

The levels of improvement during HHP processing depend on a variety of factors such as treatment conditions, type of compound, food physical characteristics, and composition. While, after HHP processing, the method used for phenolic extraction (i.e., type of solvent, ratio solvent: sample, contact, time, etc.) could influence the yield obtained among different studies from different authors. In addition, the storage conditions and handling after and during processing could influence results among different studies. As mentioned before, during processing, HHP could influence enzymes increasing their activity due to factors such as the release of membrane-bound enzymes or configuration changes, this last mechanism is also related to decreasing activity [57]. According to this, the extraction yields for the different samples could also be related to the residual activity of oxidative enzymes and the contact between phenolic and oxidative enzymes released from plant tissues after pressurization, which promotes oxidative reactions. The enzymatic activity could explain why at certain treatment conditions, no increment in phenolics was observed, but rather a decrease. Some examples are *Silvetia compressa*, where 400 MPa (15 min) and 600 MPa (5 min) decreased phenolics by 30 and 41%, respectively [72]. In cases where the change in the extractability yield of phenolics was not observed, it could be related to the retention of phenolics by the cell wall components. Some examples are gooseberry treated at 300 MPa for 5 min stored for 60 days [68], wild berry treated at 400 MPa for 20 min [77], and even cricket treated at 500 MPa for 15 min at a temperature of 65 °C [71].

It has been suggested that the increment in bioactive compounds by HHP could result in better bioavailability. Bioavailability determines the number of bioactive compounds that are digested, absorbed, and metabolized and therefore it determines their action in the human body [78,79,80]. For apple (*Granny Smith*) treated at 500 MPa, it was showing a higher absorption of minerals produced by high solubility in the intestine [79] [81], while for orange juice, higher bioavailability of vitamin C was observed. There are not enough studies showing the relationship between increment in phenolics and better bioavailability. The treatment of olives (Azeitera, Carrasqueña, Conserva de Elvas, and Morisca) at 600 MPa for 6 min at 10 °C showed an increase of bioavailability in phenols in the large intestine [79]. A review from Serment-Moreno et al. [9] concluded that HHP treatment (200–600 MPa) improved the bioavailability of phytochemical contents. This effect on the increase in bioavailability reported after HHP treatment can be related to differences in cell wall structures and improvement in the capacity of binding the phenolic compounds in the food matrix [1,4].

## 4. Final Remarks

This work describes two possible mechanisms for the increment of phenolics in foods after HHP treatment and during storage. The overview presented suggested that the increments could be related to phenolic biosynthesis and improvement of their extractability in food. The enhancement of the extraction of phenolic through HHP has been related to an immediate response to stress, where the HHP disrupts cellular compartments enhancing mass transfer and extractability or due to the disruption of non-covalent interactions between phenolics and cell wall. Despite a few cases, HHP has shown to be more effective at higher pressure levels, (>200 MPa); however, the level of yield achieved highly depends on the type of food and intensity of the other variables (time, temperature) as well as the extraction/analysis methods used. The use of HHP at lower values of pressure (<100 MPa) has been shown to activate biosynthesis of phenolic in a late response where the higher production of ROS such as H_2_O_2_ activates metabolic routes that increase the phenolic content. Despite very few cases, the use of HHP showed to be a very innovative and promising technology to improve the phenolic compounds in plants. The results presented in this review are highly relevant for the future use of HHP technology for both biosynthesis and extraction of phenolics or even other secondary metabolites with functional activities. This technology could help to generate food with better nutritional and functional value, including enhanced antioxidant activity, in addition to a better extraction could even influence the bioavailability of these compounds in the human body, increasing their beneficial effects on health.

## Figures and Tables

**Table 1:** High hydrostatic pressure effects on the biosynthesis of phenolic in different vegetable systems.

Sample	Treatment Conditions				Storage Conditions	Analyzed Compound	Main Findings		Reference
	P (MPa)	t (min)	CUT (s)	T (°C)			Approximate Change (%)	PAL Activity (%)	
Mango *Mangifera indica* (Whole fruit)	15–60	10–20	3, 10 & 28	25	2–14 days at 25 °C and 85–90% RH	Total phenols	↓7.2 up to ↑68.4	NR	[46]
						Flavonoids	↓38.6 up to ↑36.8	NR	
Mango *Mangifera indica* (Whole fruit)	20–80	10	NR	20	1–16 days at 13 °C with 85% RH	Total phenols	↓5.2 up to ↑30	NR	[47]
						Flavonoids	↓27.6 up to ↑69.7	NR	
*Vitis vinifera* (Suspension culture)	40	10	NR	25	1–7 days at 25 °C	Anthocyanin	↓53.9 up to ↑53.3	NR	[48]
Carrots *Daucus carota* (Whole vegetable)	60 & 100	CUT	15.33 & 20.67	22	0–3 days at 15 °C CO_2_ < [0.5 *v*/*v*]	Total phenols	↓11.8 up to ↑154.9	↓61.4 up to ↑380	[49]
Potato *Solanum tuberosum* (suspension culture)	100–200	10	NR	25	1–24 h	Polyphenols	↑54.0 up to ↑456.0	↑199	[44]
Strawberry *Seolhyang, Fragaria × ananassa* Duch (Whole fruit)	30–90	5	NR	25	NR	Total phenols	↑6.4 up to ↑23.1	NR	[50]
						Anthocyanin	↓16.9 up to ↑10.0	NR	

P: Pressure; t: time; T: Temperature; CUT: Come up time (time to achieve desired pressure); NR: Not reported; RH: Relative humidity. ↑ indicates an increment of content compared with the untreated sample; ↓ indicates decreasing of content compared with the untreated sample.

**Table 2:** Effect of high hydrostatic pressure on phenolic extraction yield.

Sample	Analyzed Compound	Treatment Conditions				Storage Conditions	Approximate Change (%)	Reference
		P (MPa)	t (min)	CUT (min)	T (°C)			
Apricot nectar *Prunus armeniaca* L.	TPC (Individual phenols include: Catechin, Chlorogenic acid, Neochlorogenic acid, Epicatechin, Ferulic acid, Caffeic acid, p-Coumaric acid)	300–500	5–20	2.5–4.2	34–40	2 days at 4 °C	↑2.0 up to ↑12.5	[64]
Sour cherry pomace *Prunus cerasus* L.	TPC	400 & 500	1–10	NR	20	−4 °C until analysis	↑39.5 up to ↑109.9	[65]
Grape by products (Skin, stems, and seeds) *Vitis Vinifera*	TPC	600	60	NR	70	NR	↑48.0	[66]
	Anthocyanins	600	60	NR	70	NR	↑41.4	
Jerusalem Artichoke *Helianthus tuberosus* L.	TPC (Pre-fermentation)	100	24 h	NR	50	NR	↑36.6	[67]
	TPC (Post-fermentation	100	24 h	NR	50	NR	↑61.36	
Cape gooseberry pulp *Physalis peruviana* L.	TPC	300–500	1–5	NR	25	0 and 60 days at 4 °C	↓32.3 up to ↑35.9	[68]
Grape *Vitis Vinifera*	TPPC	200–550	10	28.6 s–78.6 s	20	4 °C until fermentation(13 days)	↑55.0 up to ↑75.0	[69]
Wild Berry *Lonicera caerulea*	TPC	200–600	5–20	4–12 s	25	4 °C until analysis (48 h)	↓10.0 up to ↑14.4	[70]
	Anthocyanins	200–600	5–20	4–12 s	25	4 °C until analysis (48 h)	↓6.3 up to ↑7.9	
Açai Pulp *Euterpe oleracea Martius*	TPC	600	5	NR	25 and 65	Stored for 24 h with oxygen and light barrier	↓10.3 up to ↑11.4	[63]
Cricket *Acheta domesticus*	TPC	500	15	NR	30 and 40	NR	↑9.3 up to ↓67.3	[71]
Mealworm *Tenebrio molitor*	TPC	500	15	NR	30 and 40	NR	↓23.7 up to ↑8.6	[71]
*Silvetia compressa*	TPPC	400	15	2.03	35	Stored in brown glass flask at 10 °C	↓41.0	[72]
		600	5	3.07	35		↓30.0	
*Ecklonia arborea*	TPPC	400	15	2.03	35	Stored in brown glass flask at 10 °C	↑46.0	[72]
		600	5	3.07	35		↑20.0	
Green tea *Camellia sinensis* L.	TPC	490	15	25	25	NR	↑32.6	[73]
Longan fruit pericarp *Dimocarpus longan* L.	TPC	500	2.5	NR	30	4 °C until analysis	↑43.8	[59]
Korean barberry *Berberis koreana*	TPC	500	5 & 15	NR	25	−20 °C until analysis	↑29.9 up to ↑33.1	[74]
*Grape pomace*	TPC	50–200	5–30	NR	25	NR	↓27.9 up to ↑18.6	[75]

P: Pressure; t: time; T: Temperature; CUT: Come up time (time to achieve desired pressure); NR: Not reported; TPC: total phenolic content. TPPC: total polyphenol content; ↑ indicates the increment in content compared with the untreated sample; ↓ indicates a decrease in content compared with the untreated sample.
